# The effect of diet and time after bacterial infection on fecundity, resistance, and tolerance in *Drosophila melanogaster*

**DOI:** 10.1002/ece3.2185

**Published:** 2016-05-25

**Authors:** Megan A. M. Kutzer, Sophie A. O. Armitage

**Affiliations:** ^1^ Institute for Evolution and BiodiversityUniversity of MünsterHüfferstrasse 148149MünsterGermany

**Keywords:** Dietary manipulation, life history responses, persistent infections, plasticity

## Abstract

Mounting and maintaining an effective immune response in the face of infection can be costly. The outcome of infection depends on two host immune strategies: resistance and tolerance. Resistance limits pathogen load, while tolerance reduces the fitness impact of an infection. While resistance strategies are well studied, tolerance has received less attention, but is now considered to play a vital role in host–pathogen interactions in animals. A major challenge in ecoimmunology is to understand how some hosts maintain their fitness when infected while others succumb to infection, as well as how extrinsic, environmental factors, such as diet, affect defense. We tested whether dietary restriction through yeast (protein) limitation affects resistance, tolerance, and fecundity in *Drosophila melanogaster*. We predicted that protein restriction would reveal costs of infection. Because infectious diseases are not always lethal, we tested resistance and tolerance using two bacteria with low lethality: *Escherichia coli* and *Lactococcus lactis*. We then assayed fecundity and characterized bacterial infection pathology in individual flies at two acute phase time points after infection. As expected, our four fecundity measures all showed a negative effect of a low‐protein diet, but contrary to predictions, diet did not affect resistance to either bacteria species. We found evidence for diet‐induced and time‐dependent variation in host tolerance to *E. coli*, but not to *L. lactis*. Furthermore, the two bacteria species exhibited remarkably different infection profiles, and persisted within the flies for at least 7 days postinfection. Our results show that acute phase infections do not necessarily lead to fecundity costs despite high bacterial loads. The influence of intrinsic variables such as genotype are the prevailing factors that have been studied in relation to variation in host tolerance, but here we show that extrinsic factors should also be considered for their role in influencing tolerance strategies.

## Introduction

Higher host health is not always equated with superior pathogen resistance. If immune defenses are costly (McKean and Lazzaro 2011) and investment in defense is optimized in the face of other life history demands such as reproduction (Sheldon and Verhulst 1996), then a more resistant host may not be healthier than an individual that can reduce negative fitness effects associated with pathogenicity (Råberg et al. 2009). While host resistance measures the ability of hosts to reduce pathogen load, tolerance measures the ability of hosts to reduce the health or fitness impact of a given pathogen load (Råberg et al. 2007, 2009; Schneider and Ayres 2008; Baucom and De Roode 2011). Distinguishing between these concepts is important because of the different effects that these strategies have on the ecology and evolution of host–pathogen interactions (Råberg et al. 2007). Resistance is predicted to reduce the prevalence of a pathogen in a host population and lead to antagonistic coevolution, while tolerance will have a neutral or positive effect on pathogen prevalence (Roy and Kirchner 2000; Råberg et al. 2009). Although plant evolutionary ecologists have appreciated the concept of tolerance for many years, animal ecologists have traditionally measured resistance rather than tolerance, and it is only recently that studies have emerged on tolerance as an immune strategy in animal systems (Råberg et al. 2007, 2009; Baucom and De Roode 2011).

In the aforementioned studies, resistance is typically measured as the inverse of the magnitude of an infection, where a greater pathogen load equates to lower resistance (Råberg et al. 2009). Measures of resistance have included, for example, peak parasite density in blood (Råberg et al. 2007), whole body bacteria load (Corby‐Harris et al. 2007; Ayres and Schneider 2008, 2009), and external parasite spore load (Sternberg et al. 2012, 2013). In contrast, tolerance is measured as the slope of a reaction norm (Simms 2000), where host fitness or health is plotted against increasing parasite infection intensity for individuals from a given host type (Råberg et al. 2009). Fitness measures vary according to the study system and include longevity or survival (Corby‐Harris et al. 2007; Ayres and Schneider 2008, 2009; Lefèvre et al. 2011; Sternberg et al. 2012; Howick and Lazzaro 2014), body weight (Råberg et al. 2007; Hayward et al. 2014) and fecundity (Corby‐Harris et al. 2007; Howick and Lazzaro 2014). A flat reaction norm represents a tolerant host type that can minimize the negative fitness effects of increasing infection intensity across the observed range of pathogen load (Råberg et al. 2007, 2009). A host type with a negative slope is less tolerant because it cannot mitigate the damage caused by increasing pathogen load (Råberg et al. 2007, 2009), and a positive slope indicates over compensation in tolerance (Strauss and Agrawal 1999; Stowe et al. 2000; Jackson et al. 2014). The y‐intercept of the slope, termed general vigor, denotes uninfected host fitness (Råberg et al. 2007).

The intrinsic and extrinsic factors driving variation in tolerance in animals are not fully understood (Råberg 2014). By comparing the reaction norms of different host genotypes, it is clear that genotypic variation for tolerance does exist (e.g., Råberg et al. 2007; Regoes et al. 2014), but is not necessarily the rule (e.g., Lefèvre et al. 2011), and tolerance can vary as a function of host physiology (Jackson et al. 2014; Regoes et al. 2014). Similarly, extrinsic factors such as diet have been shown to influence tolerance (e.g., Sternberg et al. 2012; Howick and Lazzaro 2014) and other aspects of immune defense (reviewed in Ponton et al. 2013). Individuals are expected to allocate limited resources in order to maximize their reproductive success (Stearns 1992). Resource allocation and the resulting trade‐offs are central to life history theory, which predicts that hosts will reallocate resources away from traits such as reproduction or growth to survival when faced with an infection (Stearns 1992). Alternatively, a host can employ a terminal investment strategy, where resources are allocated to reproduction instead of survival or immune defense (Adamo 1999). If such trade‐offs exist, they can be difficult to detect under *ad libitum* conditions encountered in the laboratory due to compensatory resource intake and may instead become detectable under dietary restriction (Moret and Schmid‐Hempel 2000). In addition to physiological trade‐offs, resource acquisition itself can be negatively affected by infection (e.g., Ayres and Schneider 2009; Bashir‐Tanoli and Tinsley 2014). In *D. melanogaster,* dietary restriction of yeast, a major protein source, reduced resistance to *E. coli*, a nonpathogenic bacterium (McKean and Nunney 2005), but had no effect on resistance to *Providencia rettgeri* (McKean et al. 2008), whereas combined yeast and sucrose restriction had age‐dependent weak positive effects on resistance to *L. lactis* (Burger et al. 2007). However, it is unknown whether yeast restriction affects tolerance.

**Figure 1:**
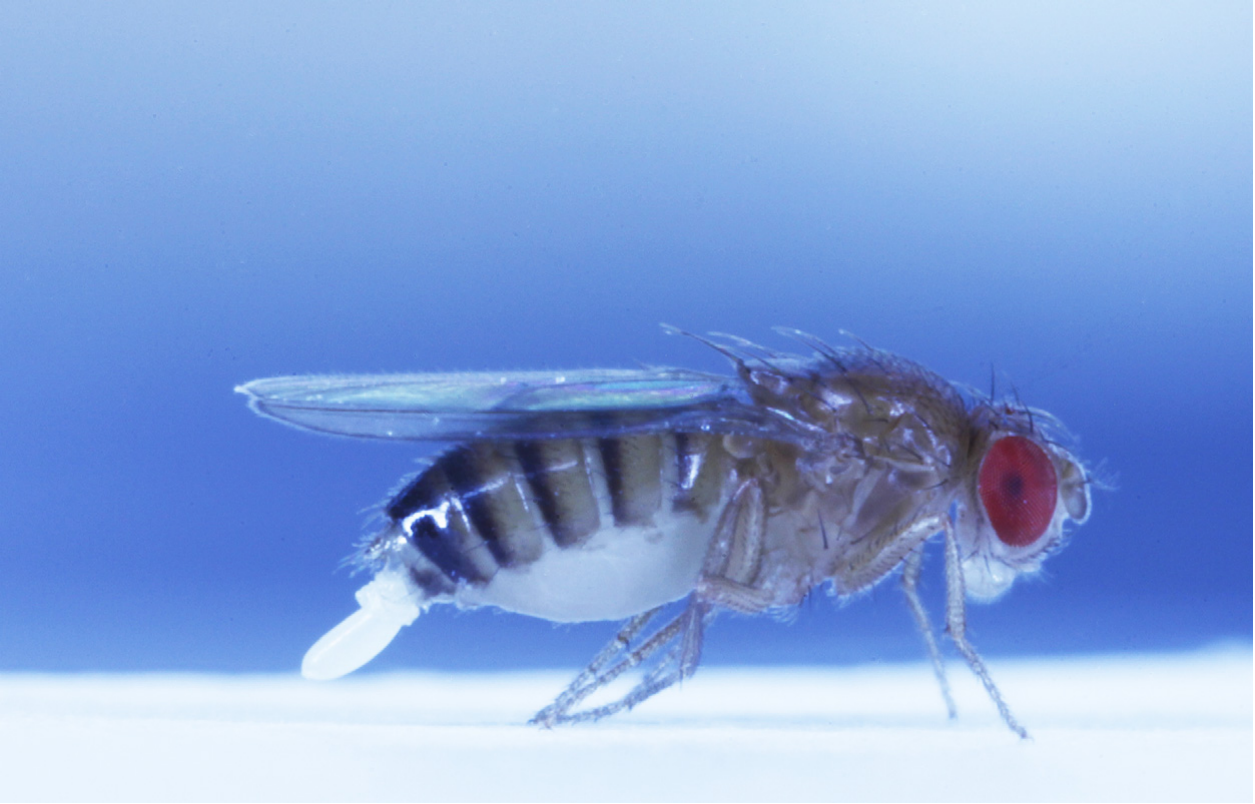
An ovipositing *Drosophila melanogaster*.

In this study, we examined the effects of dietary protein restriction on resistance and tolerance in female *D. melanogaster*. We chose two infective bacteria, *E. coli* and *L. lactis*, which cause low mortality in the time frame of this experiment, when injected into the hemocoel at the dose used. *E. coli*, a Gram‐negative bacterium*,* is comparatively non‐pathogenic but still activates the production of antimicrobial peptides (Lemaitre et al. 1997; Leulier et al. 2000; Armitage et al. 2014). *L. lactis,* a Gram‐positive, opportunistic pathogen, was isolated from the hemolymph of wild‐caught *D. melanogaster* (Lazzaro 2002; Lazzaro et al. 2006) and is known to result in comparatively high bacterial loads and low mortality 28 h post infection (HPI) (Lazzaro 2002; Lazzaro et al. 2006). Although neither bacteria are obligate pathogens, we reasoned that tolerance could be measured at bacterial loads that are experimentally detectable but non‐lethal to their host because host mortality would make quantification of fecundity and infection intensity unreliable in the absence of information on the precise time of death. The dynamics of resistance and tolerance may be expected to change over the course of the infection (Hayward et al. 2014; Howick and Lazzaro 2014); therefore, we chose two acute infection phase time points (24 and 72 h) to assay bacterial load (the inverse of which is resistance) and fitness. The importance of examining host responses at different timepoints after infection was underlined by a recent study on individual infection trajectories in mice (Lough et al. 2015). Individual mice that survived an infection exhibited a typical and reproducible pattern in their trajectories. In this case, resistance was important early in the infection and tolerance, later in the infection. We measured fecundity as the number of eggs laid (Fig. 1) up to 72 h postinfection, the number of adult offspring that eclosed from these eggs, and egg to adult viability, and in a second experiment, we assayed egg quality, measured as total protein content (Ahmed et al. 2002; Reaney and Knell 2010; Stahlschmidt et al. 2013). While previous studies on *D. melanogaster* have examined intergenotype variation and group means to estimate tolerance (Corby‐Harris et al. 2007; Ayres and Schneider 2008; Howick and Lazzaro 2014), here we estimate variation within a single genotype (e.g., Sternberg et al. 2012) by measuring fitness and bacterial load from the same individuals and then determining tolerance slopes for each of our treatment groups (Råberg et al. 2007, 2009; Graham et al. 2011; Lefèvre et al. 2011).

## Materials and Methods

### *Drosophila melanogaster* culture conditions

The wild‐type stock used in the study originated from ten inseminated females that were wild‐caught at several locations in Münster, Germany, in 2008. The stock was maintained in a population cage containing overlapping generations and kept at 25°C, 70% relative humidity on a 12‐12 h light–dark cycle. Flies were kept on a standard sugar, yeast, agar medium containing 1.5% agar, 5% sugar, 10% yeast, 3% nipagin and 0.3% propionic acid (SYA medium) (Bass et al. 2007).

#### Experiment 1: The effect of diet, bacterial infection species, and time after infection on fecundity, resistance, and tolerance

The procedures described below were repeated three times to produce three experimental replicates.

### Experimental animals and dietary treatments

The flies used for both experiments, as well as their parents, were reared at constant larval density: 4 weeks prior to infections, we placed a grape juice plate supplemented with fresh yeast paste in the population cage for embryo collection. Flies were allowed to oviposit for 8 h. Then the plate was removed from the cage, and the wet yeast was removed. The grape juice plate was incubated at 25°C for 24 h. After 24 h, nearly all embryos had hatched and 2000 first instar larvae were collected and placed into glass vials (100 × 25 mm) containing SYA medium. Each vial contained 100 larvae (F1 generation), which were allowed to develop into adults. Thirteen days later, flies from the F1 generation were placed in embryo collection cages (flystuff.com) in groups of approximately 500 on a grape juice agar plate supplemented with wet yeast. F1 flies were allowed to oviposit for 8 h and then were discarded. Approximately 2000–3000 first instar larvae were collected from the plates and placed in groups of 100 on SY medium. After eclosion, experimental virgin male (*n* = 300 for each experimental replicate) and female (*n* = 300) F2 flies were collected and separated into groups of twenty under light CO_2_ anesthesia. Female flies were assigned to one of two diets, either the standard SYA medium (hereafter, SY) or a reduced medium (RY) containing 2.5% yeast, that is, 25% of the yeast contained in the SY medium (Fricke et al. 2008). All male flies were kept on SY medium. F2 flies were kept at 25°C, 70% relative humidity on a 12‐12 h light–dark cycle, and aged 8 days prior to mating.

### Mating assay

Both the mating assay and the bacterial infections were performed at room temperature. Each female was allowed to copulate once. Approximately 18 h before mating, 300 virgin males were placed into individual vials: 150 on SY medium and 150 on RY medium. The following morning, individual females on corresponding food treatments were transferred to a vial containing a male. The time the female entered the vial was recorded, and copulations were observed and the start and finish time recorded. Each fly was given a maximum of 2 h to mate. Any flies mating for <5 min were discarded, as this likely means that sperm were not transferred (Gilchrist and Partridge 2000). Males were removed from each vial immediately following copulation and discarded to prevent remating.

### Bacterial preparation and infections

Aliquots of *E. coli* (K12 wildtype, DSM no. 498, German collection of microorganisms and cell cultures [DSMZ]) and *L. lactis* (gift from Brian Lazzaro) stored at −80°C were plated out on lysogeny broth (LB) agar (4 agar plates per bacteria species) and incubated at 30°C for 24 h. One clone from each plate was picked into 100 mL of sterile LB broth in an Erlenmeyer flask (4 clones per 100 mL culture) and left to grow overnight (approximately 15 h) at 30°C, 200 rpm. The next morning, the bacteria cultures were centrifuged at 2880 rcf at 4°C for 10 min, after which the supernatant was removed. The bacteria were washed two times in *Drosophila* Ringer's solution (182 mmol·L^−1^ KCl; 46 mol·L^−1^ NaCl; 3 mmol·L^−1^ CaCl_2_; 10 mmol·L^−1^ Tris·HCl; Werner et al. 2000) using 2880 rcf at 4°C for 10 min after each washing step. The bacterial solution was counted and adjusted to a concentration of 1 × 10^8^ cells·mL^−1^. Each bacterial solution was kept on ice throughout the injections.

Injections were performed 28 ± 1 h after mating using a randomized block design for the eight treatments (diet followed by infection treatment): SY‐Naïve, SY‐Ringer's, SY‐*E. coli*, SY‐*L. lactis*, RY‐Naïve, RY‐Ringer's, RY‐*E. coli*, RY‐*L. lactis*. Females were anesthetized individually using light CO_2_. One hundred and sixty flies (480 flies in total from the three experimental replicates, giving *n* = 30 flies per treatment group) were injected in the lateral side of the thorax using a fine glass capillary attached to a Nanoject II^™^ (Drummond). Flies were injected with 18.4 nL of *E. coli* or *L. lactis,* yielding a dose of approximately 1840 bacteria per individual. In preliminary experiments, we found that this dose resulted in approximately zero mortality for *E. coli‐* and 10% mortality for *L. lactis*‐infected flies, 72 HPI, while yielding measurable bacterial loads. The wounded control group received 18.4 nL sterile *Drosophila* Ringer's solution. The naïve treatment groups were only subjected to light anesthesia with CO_2_. Post‐infection, flies were returned to 25°C, 70% relative humidity on a 12‐12 h light–dark cycle. The injected solutions were checked for contamination and bacterial concentration. After injections, the remaining Ringer's and bacteria‐Ringer's mixtures were plated out onto LB agar plates and incubated for 24 h at 30°C to check for environmental contamination. We found no evidence of alien bacteria/fungi from any of the plated solutions. We also diluted the bacteria aliquots to 1 × 10^3^ cells·mL^−1^ and 100 *μ*L of each solution was plated out and incubated for 24 h to confirm the concentration of the injection dose. All plates should have had 100 CFUs on them. We counted between 27 and 83 CFUs for *L. lactis* over the three replicates and between 107 and 123 CFUs for *E. coli* over the three replicates. Bacteria loads in the flies were not correlated with the aliquot counts.

### Fitness measures

Females were placed into new food vials (SY or RY depending upon treatment) every 24 h for 72 h. This allowed us to count the number of eggs laid per female 24, 48, and 72 (±2) HPI. After egg counting, each vial was incubated at 25°C until the offspring had completed development. The diet affected developmental time; therefore, offspring kept on SY medium were given 12 days and those kept on RY were given 17 days to complete development. All adults were counted as our second measure of fecundity (adult offspring number), and our third measure, egg to adult viability (shortened hereafter to viability), was calculated as the proportion of eggs that developed to adults.

### Resistance measure

We assayed bacterial load at 24 and 72 (±1) h post‐infection to quantify resistance (inverse of load). We sacrificed 80 animals per experimental replicate at each timepoint: 10 animals from each of the eight treatment groups. Each fly was anesthetized, dipped in 1 mL of 70% ethanol and then 1 mL of sterile water and placed in a 1.5 mL microcentrifuge tube containing a sterile tungsten carbide bead (Qiagen) and 200 *μ*L of LB broth on ice. Flies were homogenized for 30 sec at a frequency of 20 Hz using a Retsch Mill (MM301). Whole fly homogenates from those infected with *E. coli* were serially diluted from 1:1 to 1:10, and homogenates from flies infected with *L. lactis* were serially diluted from 1:1 to 1:1000. We plated 100 *μ*L of each whole fly homogenate and dilution(s) onto LB agar and incubated the plates at 30°C for 20 h, when we counted colony‐forming units (CFUs) to obtain an approximate bacterial load for each individual fly. Individuals whose homogenate contained more CFUs were considered to be less resistant and vice versa. If a plate contained too many CFUs to count at the highest dilution, we assigned the value as the greatest number of CFUs that had been counted in that treatment group/across the whole experiment (*e.g.,* Vincent and Sharp 2014), although this is likely to be an underestimate. Homogenates from three individuals in the *L. lactis* treatment groups at 72 HPI contained too many CFUs to count at any dilution. Approximately 7% of the homogenates across all treatment groups contained CFUs with a foreign morphology (i.e., not *E. coli* or *L. lactis*) or contained CFUs when they should not have (Ringer's and naïve groups). In these cases, we removed these individuals from our analyses.

#### Experiment 2: The effect of diet and bacteria infection species on egg protein content and bacterial persistence in the fly

We assayed egg protein content to test for trade‐offs in response to infection using the diet, mating, and infection setups described for Experiment 1. For each of the eight treatment groups, we injected 80 females (320 animals in total), and the experiment was performed once. We collected eggs from the vials of all individuals that had laid four eggs or more between 24 and 44 HPI (37.5% individuals on RY and 73% on SY). Eggs start to hatch after 20 h; therefore, we did not collect eggs at 48 HPI. Preliminary experiments showed that four was the minimum number from which we could obtain reliable protein measures using a Bradford assay. The eggs were washed twice in 100 *μ*L phosphate‐buffered saline (PBS, pH 7.2) to remove food traces and then placed in 55 *μ*L of PBS on ice. Each sample was sonicated on ice using a Bandelin Sonoplus on 25% power in five, 1 sec bursts every 30 sec. Each sample was spun down for 1 min at 1699 rcf and then checked under a microscope to ensure that there were no visible egg fragments. The samples were kept frozen at −20°C for maximum 4 days. We diluted each sample 1:2 in PBS and quantified protein using Roti^®^‐Nanoquant solution according to the manufacturer's instructions in 96‐well culture plates with the following modifications: The calibration standard was prepared in PBS instead of ddH_2_O, and each sample was run in duplicate on a Tecan plate reader. We divided the total concentration of each protein solution by four to obtain average protein per egg in *μ*g. In addition, we kept all flies on their respective diets, checking their survival daily until 168 HPI when we homogenized fifteen individual flies from each treatment group, in 200 *μ*L LB broth. We plated out 100 *μ*L of the whole fly homogenate to determine whether flies had cleared the bacteria by this later timepoint, incubated the plates at 30°C, and counted the CFUs 20 h later.

### Statistical analyses

Statistical analyses were performed in R 3.2.2 (R Core Team 2015). Linear mixed effects models and generalized linear mixed models were analyzed using the glmer function in the lme4 package (Models 1a,b and 2) (Bates et al. 2015) or the lme function (Models 3–6), available in the nlme package (Pinheiro et al. 2015). The statistical outputs were comparable using these functions, the lmer function in lme4 in the case of the linear mixed effects models, or when using a penalized quasilikelihood approach, available in the MASS package in the case of generalized linear mixed models (Ripley et al. 2015). We tested for main effects using Wald F or *χ* ^2^ tests (Bolker et al. 2009). In cases where the residual spread differed by HPI and/or Diet (e.g., models 3, 4, and 6), we used the varIdent function in the nlme package to correct for this by specifying that there were differences in the standard deviation among our grouping factors (e.g., Diet × HPI or Diet) (Zuur et al. 2009). The main models are detailed below, and model parameter estimates and standard errors are listed in Tables S1–S4. The models and results for mating latency, copulation duration, and fecundity read outs for animals homogenized at 24 HPI from Experiment 1 are in Appendix S1 and Table S5, respectively. Raw data is available in the Dryad database.

#### Experiment 1: Effect of diet and infection on fitness

We tested for changes in fecundity due to diet and infection treatment over the course of an infection using generalized linear mixed models in the lme4 package with Poisson or binomial error structures. Fecundity was measured as the number of eggs laid in each 24‐h period, the number of eclosed adult offspring, or the proportion of eggs completing development to adults (viability). The fixed factors were infection status (i.e., naïve, Ringer's, *E. coli,* or *L. lactis*), diet (i.e., SY or RY), and hours post‐infection (HPI; i.e., 24, 48, or 72 h postinfection). We started with the full model in all cases: Infection status*Diet*HPI and evaluated reduced model likelihoods with AIC scores. We included Fly ID and Run ID (i.e., a different number was given for each repeated measure from every individual) as random effects to control for pseudoreplication (Fly ID) and overdispersion (Run ID) (Harrison 2014). Blocks nested within experimental replicates were included as random effects. We obtained similar results when both response variables were natural log‐transformed, which omitted the need for an observation‐level random effect. Egg and adult offspring numbers were modeled with Poisson errors and egg viability with binomial errors. The residuals showed signs of non‐normality but we were confident in the model output because our observation level random effect controlled for inflated *P*‐values. After model reduction, the models explaining significant variance in fecundity, tested as eggs, offspring, or viability, were as follows:

Model 1a, b: Fecundity_Eggs or Offspring_ ˜ Infection status + Diet + HPI + Infection status * HPI + Diet * HPI + Replicate/Block/FlyID/RunID_random_
Model 2: Egg viability ˜ Infection status + Diet + HPI + Infection status * Diet + Diet * HPI + Replicate/Block/FlyID/RunID_random_

#### Experiment 1: Effect of diet and infection on bacterial load

We investigated whether there was a significant effect of diet and HPI on host resistance to bacterial infection. Bacterial load (CFU) was natural log transformed, giving reasonably normally distributed residuals. Blocks nested within experimental replicate were included as random effects. No model reduction was possible; thus, the model used to test for differences in CFU, tested as *E. coli* (3a) and *L. lactis* (3b), was the following:

Model 3a, b: CFU ˜ Diet + HPI + Diet * HPI + Replicate/Block_random_

#### Experiment 1: Effect of diet and infection on tolerance

Models 4a and b evaluated changes in fecundity tolerance over the course of the two infections and in response to dietary restriction. Our fecundity response variable (percent difference in adult offspring number) was calculated by subtracting adult offspring resulting from infected flies (infected fecundity, *ω*_*i*_) at 24 or 72 h from mean adult offspring in our wounded control (i.e., uninfected fecundity, *ω*_*0*_) for the same timepoint, and dividing the resulting value by *ω*_*0*_ and multiplying it by 100. Calculations were performed within dietary treatment. We reasoned that using the uninfected control group was more appropriate than using pre‐infection fecundity for each individual because singly mated, 10‐day‐old flies from our stock population generally lay around 20% more eggs 1 day post‐mating, that is, pre‐infection in this experiment, compared to 2 days post‐mating, that is, 1 day post‐infection (personal observation). Consequently, using pre‐infection fecundity as our uninfected y‐intercept would have skewed our results. Furthermore, standardizing the change in fitness by using percent change means that the values are more comparable across dietary treatments. We used adult offspring as our fecundity measure in the tolerance models because the number of offspring that survive to adulthood ultimately determines organismal fitness. We included natural log‐transformed bacterial load as a covariate in these models and produced a separate model for each bacterium. We started with models containing the 3‐way interactions between CFU * Diet * HPI. In the case of Model 4b, removal of the nonsignificant interaction term CFU × HPI resulted in heterogeneous residual plots, so the term was left in the model. However, the statistical results were qualitatively similar whether or not this term or the three‐way interaction were removed. This resulted in the following final models:

Model 4a: Percent Change_*E. coli*_ ˜ CFU * Diet * HPI + Replicate/Block_random_
Model 4b: Percent Change_*L. lactis*_ ˜ CFU + Diet + HPI + CFU * HPI + Diet * HPI + Replicate/Block_random_

#### Experiment 2: Effect of diet and Infection on egg protein content

We evaluated one model to examine the effect of diet and infection status, and their interaction, on egg protein content. Plate refers to the 96‐well plate on which the sample was run. Model reduction was performed as described above.

Model 5: Protein ˜ Infection status + Diet + Plate_random_

#### Experiment 2: Effect of Diet on bacterial load 168 h post‐infection

Models 6a and b evaluated the effect of diet on an *E. coli* or *L. lactis* infection after 168 h.

Model 6a,b: CFU ˜ Diet + Block_random_

## Results

### Experiment 1: Effect of diet, HPI, and infection on fecundity

**Figure 2:**
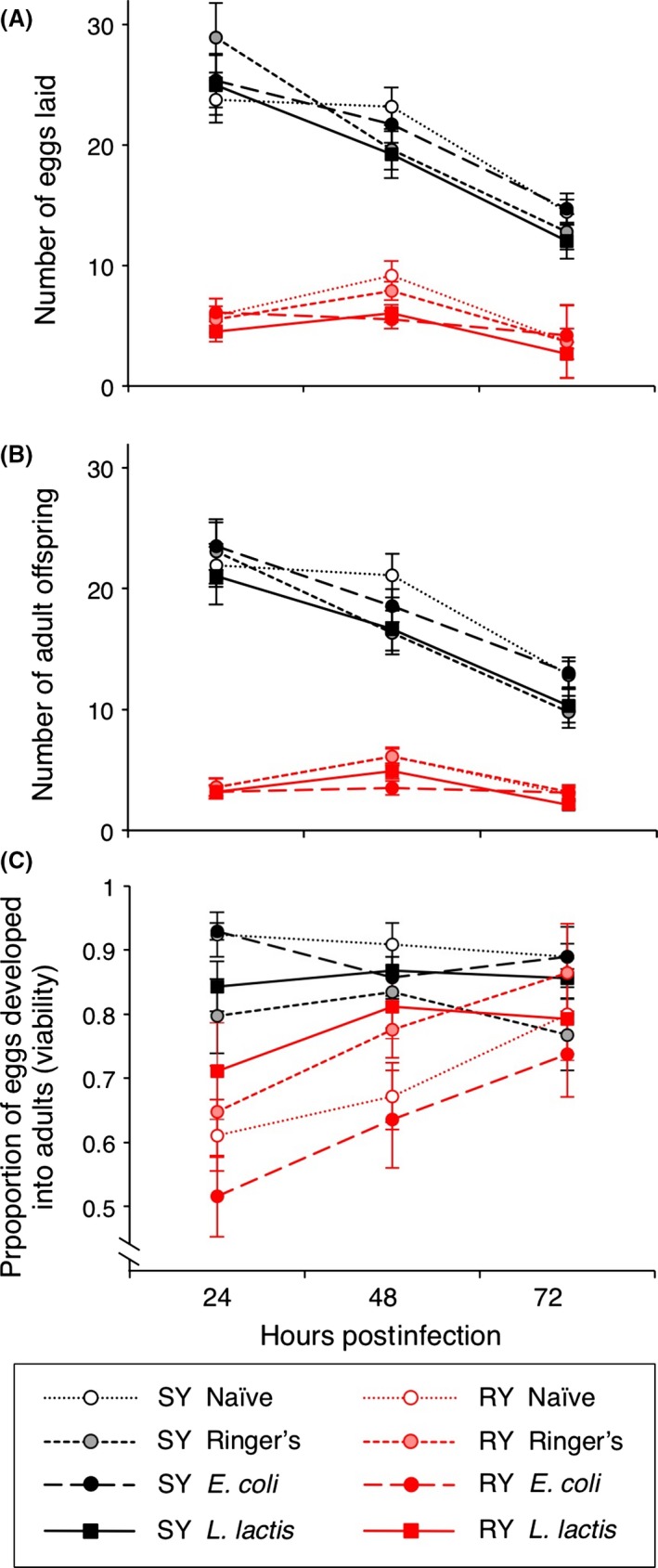
Female fecundity differed in response to diet and bacterial infection over a continuous 72‐h period post‐infection. Fecundity was measured as (A) eggs laid per 24‐h period, (B) adult offspring, and (C) the proportion of eggs that completed development to adults (viability). SY indicates the standard yeast diet and RY the reduced yeast diet. Each mean is calculated from the eggs and offspring of between 27 and 30 flies. Error bars show 1 SE. For statistics, see Table 1.

There was a significant interaction between diet and HPI (Table 1), where flies reared on the SY diet reduced egg and offspring production over time, yet the flies on the RY diet showed steady offspring production (Fig. 2A,B). The significant interaction between HPI and infection on offspring (Table 1) shows that the infection treatments responded differently, in a time‐dependent manner. Females reared on the RY diet laid significantly fewer eggs and had fewer offspring than females reared on the SY diet (Fig. 2A,B; Table 1; see Fig. S1A,B and Table S5 for fitness of flies homogenized at 24 h post‐infection).

**Table 1:** The effect of infection status, diet, and hours post‐infection (HPI) on fecundity (*n*_total_ = 240). Fecundity was measured as eggs laid per day (Model 1a), offspring per day (Model 1b), and egg to adult viability (Model 2). A dash (–) indicates that this interaction term was removed during model simplification. Values in bold are statistically significant

Tested effect	Model 1a: Eggs			Model 1b: Offspring			Model 2: Viability		
	df	*χ* ^2^	*P*	df	*χ* ^2^	*P*	df	*χ* ^2^	*P*
Diet	1	273.95	**<0.0001**	1	193.03	**<0.0001**	1	17.61	**<0.0001**
HPI	2	132.60	**<0.0001**	2	171.25	**<0.0001**	2	3.05	0.217
Infection status	3	5.06	0.168	3	2.61	0.456	3	1.46	0.692
Diet × HPI	2	29.06	**<0.0001**	2	52.76	**<0.0001**	2	10.05	**0.007**
Diet × Infection status		–	–		–	–	3	12.04	**0.007**
HPI × Infection status	6	10.88	0.092	6	13.35	**0.038**		–	–

The proportion of eggs that developed into adults (viability) showed a significant interaction between diet and HPI (Fig. 2C; Table 1), where the viability of eggs kept on standard medium was relatively steady, while the flies kept on reduced medium showed an increase in viability over time. There was also a significant interaction between diet and infection (Table 1; Fig. S2). The RY diet resulted in significantly reduced egg to adult viability compared with the SY diet for the flies homogenized at 24 h post‐infection (Fig. S1C; Tables S1).

### Experiment 1: Bacterial infection profiles differ and are not significantly affected by diet

**Figure 3:**
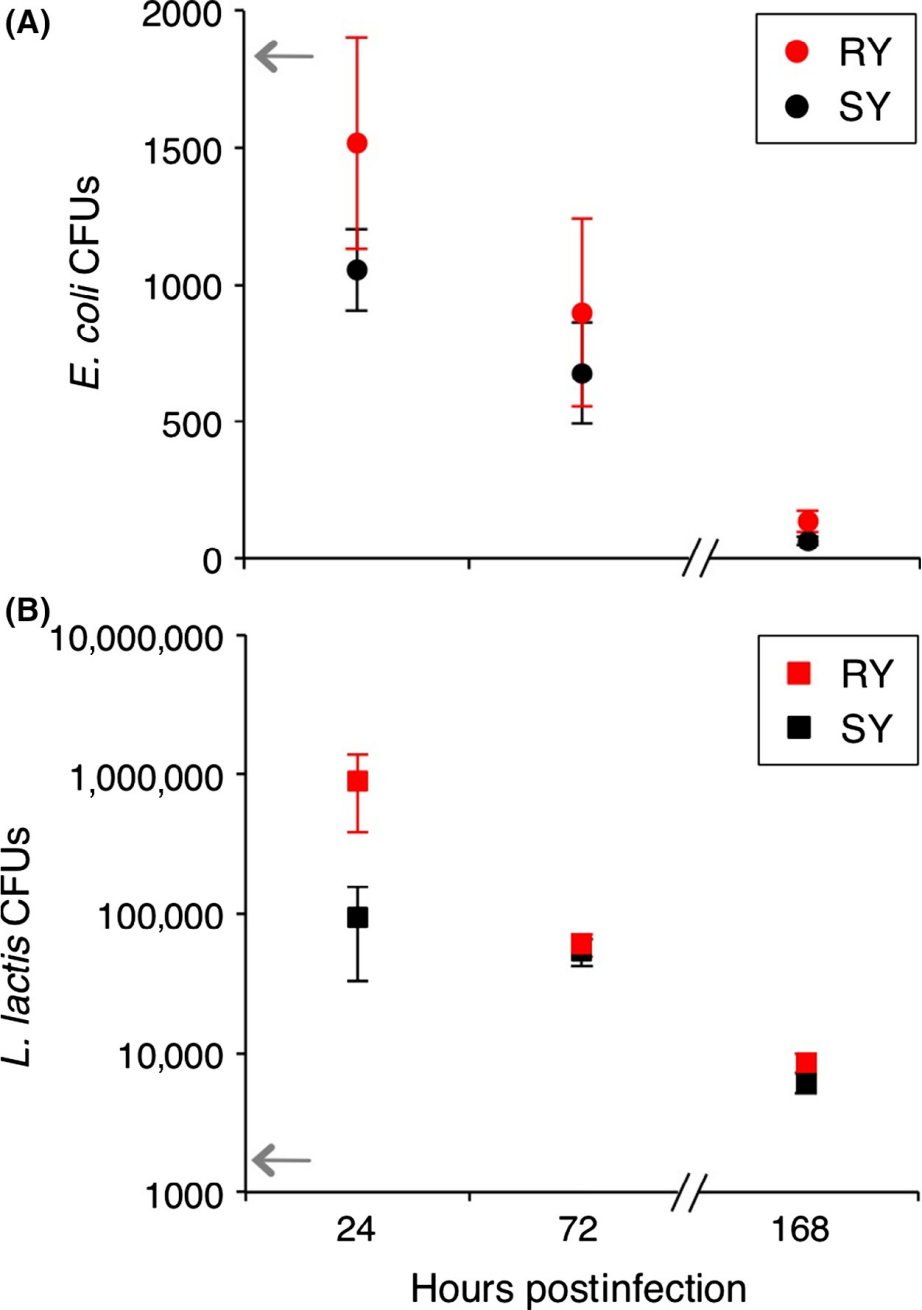
Bacterial load was affected by time post‐infection and not diet. Bacterial loads of (A) *E. coli* and (B) *L. lactis* were quantified as colony‐forming units (CFU), for 24 and 72 h (experiment 1) and 168 h (experiment 2) post‐infection. RY indicates reduced yeast diet and SY the standard diet. Each mean is calculated from the CFU counts of between 21 and 30 female flies. Error bars show 1 SE. For statistics, see Table 2. The gray arrows indicate the approximate initial injection doses.

Bacteria load was not significantly affected by diet (Table 2; Fig. 3) and decreased from 24 to 72 h post‐infection (Fig. 3; Table 2) in flies infected with both bacteria species. The reduction over time was similar for both diets (non‐significant interaction term, Table 2). Twenty‐four HPI, *E. coli‐*infected flies had average loads that were two to three orders of magnitude lower than *L. lactis*, and a similar disparity in load remained 72 HPI.

**Table 2:** The effect of diet and HPI on bacterial load. Bacterial load was measured as colony‐forming units (CFU) per fly on day 1 and day 3 postinfection. Model 3a tested differences in *Escherichia coli* load (*n*_total_ = 116) and Model 3b tested *Lactococcus lactis* load (*n*_total_ = 112). Values in bold are statistically significant

Tested effect	Model 3a: *E. coli*				Model 3b: *L. lactis*			
	*numDF*	*denDF*	*F*	*P*	*numDF*	*denDF*	*F*	*P*
Diet	1	97	1.407	0.238	1	93	0.274	0.602
HPI	1	97	16.115	**0.0001**	1	93	14.596	**0.0002**
Diet × HPI	1	97	0.387	0.535	1	93	0.361	0.549

### Experiment 1: Tolerance varies according to bacteria species

**Figure 4:**
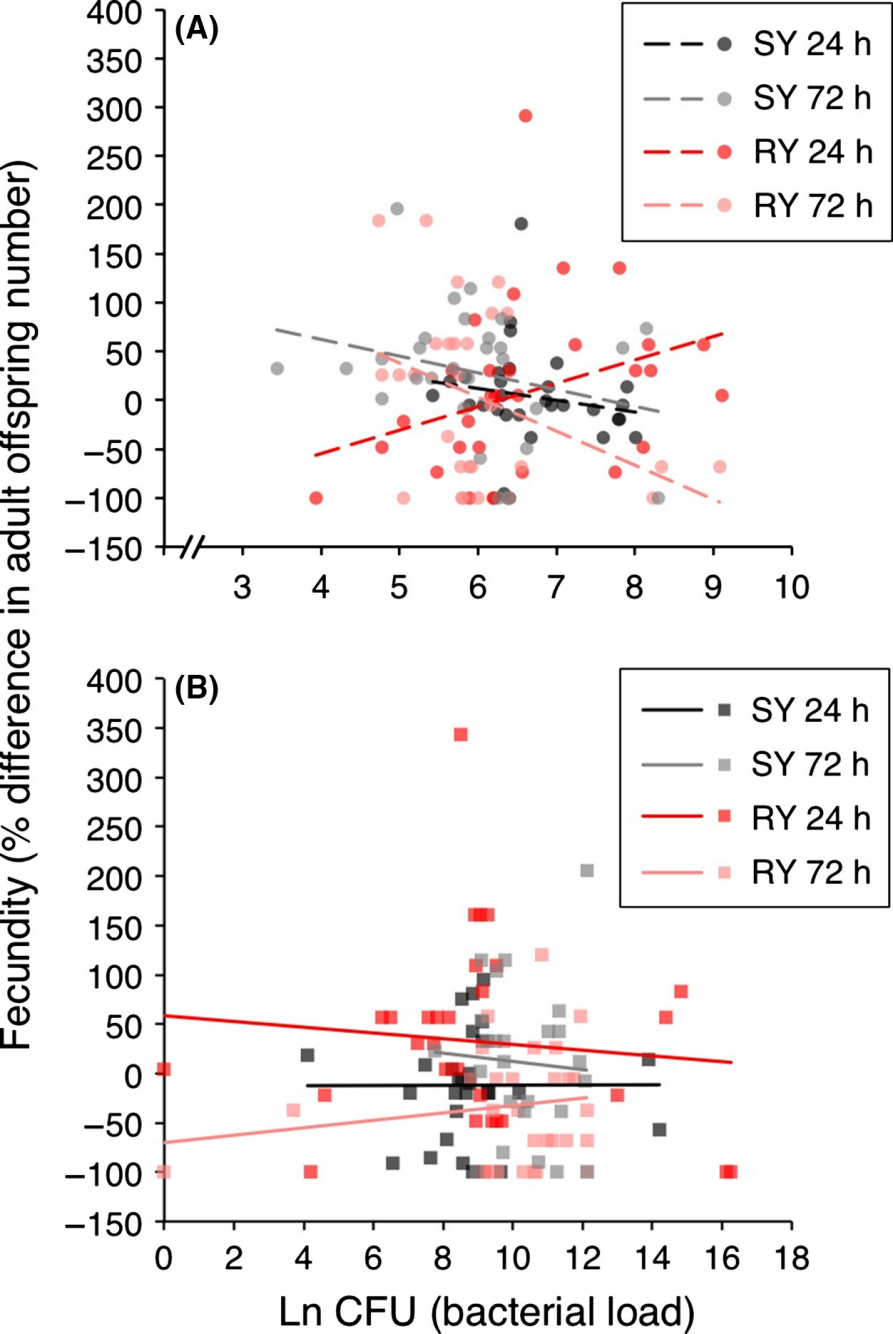
Tolerance after *E. coli* infection varies by diet and day post‐infection. Tolerance curves are plotted for (A) *E. coli* and (B) *L. lactis* on SY and RY at 24 and 72 h post‐infection. The natural log of bacterial load (CFUs) is plotted on the *x*‐axis, and the fecundity measure, percent difference in adult offspring number, is given on the *y*‐axis. The fecundity measure was calculated within diet treatment groups as follows: ((infected fecundity, *ω*_*i*_) ‐ (uninfected fecundity *ω*_*0*_))/*ω*_*0*_) × 100. Each data point gives the bacterial load and fitness of one individual female. For statistics, see Table 3.

Both diet and time after infection differentially affected fecundity tolerance in flies infected with *E. coli*, which is indicated by the significant three‐way interaction among bacterial load, diet, and HPI (Fig. 4A, Table 3); however, this is a marginal effect (*P* = 0.026). Flies on the RY diet were more tolerant than those on the SY diet at 24 HPI, but less tolerant at 72 HPI. Despite high bacteria loads, flies infected with *L. lactis* did not exhibit variation in tolerance and no effect was uncovered with food limitation. *L. lactis* infections occurring at this infection dose seem to have no effect on our measures of host fecundity, rather changes in fecundity are due to the combined effects of diet and HPI (Fig. 4B, Table 3, significant interaction Diet × HPI).

**Table 3:** The effects of bacteria load, diet, and HPI on fecundity tolerance (Adult offspring percent change) to *Escherichia coli* (*n*_total_ = 116) and *Lactococcus lactis* (*n*_total_ = 112). A dash (–) indicates that this interaction term was removed during model simplification. For model 4b, the model fit was better if the nonsignificant interaction between CFU and HPI was left in the model. Values in bold are statistically significant

Tested effect	Model 4a: *E. coli*				Model 4b: *L. lactis*			
	*numDF*	*denDF*	*F*	*P*	*numDF*	*denDF*	*F*	*P*
CFU	1	93	3.241	0.075	1	91	0.000	0.995
Diet	1	93	0.294	0.589	1	91	0.528	0.469
HPI	1	93	1.786	0.185	1	91	1.276	0.262
CFU × Diet	1	93	1.689	0.197	–	–	–	–
CFU × HPI	1	93	3.861	0.052	1	91	0.601	0.440
Diet × HPI	1	93	1.583	0.212	1	91	9.794	**0.002**
CFU × Diet × HPI	1	93	5.061	**0.027**	–	–	–	–

### Experiment 2: Diet, but not infection, affects egg protein content, and both bacterial infections are persistent

**Figure 5:**
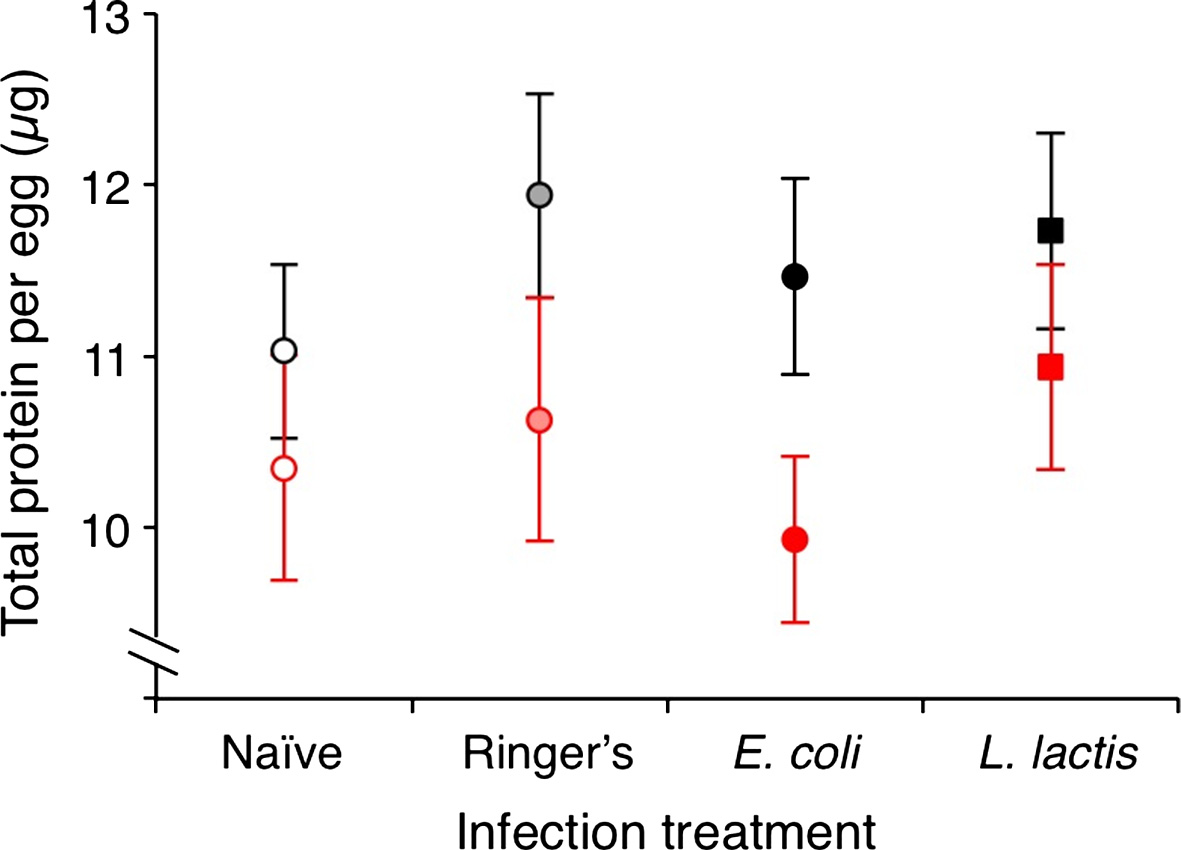
Diet, not bacterial infection, affects egg protein content. Total protein per egg (*μ*g) was calculated from four eggs per female (*n* = between 12 and 28 females per treatment) laid between 24 and 44 h postinfection. Black‐outlined symbols indicate the standard yeast diet (SY), and red‐outlined symbols indicate the reduced yeast diet (RY). Error bars show 1 SE. For statistics, see Table 4.

Reducing dietary yeast by 75% significantly reduced egg protein content compared to the standard diet (marginal significance, *P* = 0.024); however, bacterial infection had no effect on egg protein content (Fig. 5; Table 4). There was a significant effect of infection treatment on survival (*χ* ^2^ = 23.194, *P* < 0.0001; Fig. S3): The naïve, Ringer's, and *E. coli‐*infected flies had over 95% survival over the 168‐h infection period, but the *L. lactis* flies showed reduced survival. Notably, neither flies infected with *E. coli* nor *L. lactis* were able to clear the infection by 168 HPI (Fig. 2A,B, respectively) and *L. lactis‐*infected flies sustained loads well above the inoculation dose for the duration of the 168 h.

**Table 4:** The effect of diet and infection status on total egg protein (*n*_total_ = 171) (Model 5) and the effect of diet on bacterial load 7 days postinfection (*n*_total_ = 30) (Models 6a & 6b). Values in bold are statistically significant

Tested effect	Model 5: Protein				Model 6a: *Escherichia coli*				Model 6b: *Lactococcus lactis*			
	*numDF*	*denDF*	*F*	*P*	*numDF*	*denDF*	*F*	*P*	*numDF*	*denDF*	*F*	*P*
Diet	1	159	6.04	**0.015**	1	21	1.24	0.278	1	20	0.098	0.757
Infection status	3	159	1.44	0.233			–	–			–	–

## Discussion

We examined the effect of dietary yeast restriction on fecundity, resistance, and tolerance upon intrahemocoelic injection of *D. melanogaster* with two bacteria species. Bacterial loads were particularly high in the *L. lactis* treatment group, yet reproductive fitness seemed unaffected even under dietary restriction. Moreover, we uncovered diet‐ and time‐induced variation for tolerance to an *E. coli* infection but not to a *L. lactis* infection. Interestingly, flies on the RY diet were more tolerant to an infection with increasing *E. coli* loads than were flies on the SY diet 24 HPI but were less tolerant to an infection 72 HPI, indicating that tolerance can change over time and also in response to environmental factors like host diet.

### Effect of diet, HPI, and infection on fecundity

Protein availability is a major determinant of egg production in *D. melanogaster* (Drummond‐Barbosa and Spradling 2001). Complete protein restriction effectively arrests vitellogenesis at its onset (Drummond‐Barbosa and Spradling 2001). As such, yeast limitation was predicted to have an effect on fecundity. Indeed, we found a close to four‐fold increase in total egg numbers over the 3‐day experimental period in the SY (3‐day total mean = 59.9 ± 0.8) compared to the RY (3‐day total mean = 15.9 ± 2.2) diet. These values fall within the range of previous yeast limitation studies (2.5‐fold: McKean et al. 2008; 60‐fold: Drummond‐Barbosa and Spradling 2001). Perhaps unsurprisingly, reduced dietary protein negatively affected adult offspring numbers, and interestingly, also viability. Manipulation of dietary protein has previously been shown to affect egg to adult viability in evolved *D. melanogaster* lines selected on a standard or a protein‐enriched diet. However, in this example, the flies had reduced viability in the protein‐enriched diet (Kristensen et al. 2011); thus, in both this and our experiments, standard yeast concentration results in higher viability. Viability showed plasticity over time in our RY group, whereby it increased over the 3‐day experimental period. It is unclear why this might change over time but lower viability could be due to a number of factors, including less efficient egg fertilization or interference with developmental processes due to the lower protein maternal diet and subsequently, offspring diet.

We predicted that bacterial infection would result in reduced fecundity, as demonstrated in a previous study where *D. melanogaster* fecundity was reduced after injection with live and dead *E. coli* (Bashir‐Tanoli and Tinsley 2014); however, we found no such effect. We also found no effect of bacterial infection on fecundity measured as eggs and adult offspring under dietary yeast restriction, but viability showed a different response. Linder and Promislow (2009) observed that maternal infection status had no effect on egg to adult viability in flies under *ad libitum* conditions, but they proposed that resource limitation may uncover such an effect. Indeed, we found an interaction between infection status and diet, driven partly by the increase in viability of eggs from *E. coli*‐infected flies and also by an increase in viability of naïve flies kept on SY compared to RY. The fact that viability responds differently to our treatments compared to egg and offspring numbers suggests the importance of measuring more than one aspect of fecundity and might indicate trade‐offs between different fecundity traits.

In experiment two, we reasoned that the cost of a bacterial infection could also manifest itself via deposition of protein into eggs and that infected flies reared on reduced medium might adjust reproductive investment, resulting in a change in total egg protein. Stahlschmidt et al. (2013) observed such a phenomenon in the Texas field cricket: Heat‐killed bacteria chronically activated the immune system of the crickets, resulting in lower egg protein content (Stahlschmidt et al. 2013). Likewise, in mosquitoes, total ovarian protein content decreased during immune activation with LPS (Ahmed et al. 2002). However, our infection treatments did not have a significant effect on the total egg protein content in those females that had laid more than four eggs. Nonetheless, eggs from flies reared on the RY diet contained on average 9.5% less protein than eggs from flies reared on the SY diet. In experiment one, egg to adult viability was lower in flies reared on RY compared to the SY flies (*χ* ^2^ = 7.58, *P* = 0.006) over a similar, but not identical time frame (experiment 1: 24–48 h; experiment 2: 24–44 h). It would be informative to test whether lower protein content contributed toward lower viability, and more generally, whether there are other fitness consequences of reduced protein for the offspring.

### Bacterial infection profiles differ; infection is persistent and is not significantly affected by diet

Our study uncovered considerable individual variation in resistance, indicating that population‐level means may fail to reveal the full extent of variation in immune related traits; such variation may influence range‐ rather than point‐tolerance estimates. Range‐tolerance estimations take into account fitness across a range of pathogen loads, whereas point‐tolerance estimations assess mean fitness plotted against mean pathogen load (Little et al. 2010). In our study, *E. coli* and *L. lactis* displayed remarkably different infection dynamics. While both bacteria showed significantly reduced loads over time, the *E. coli* load was lower than the injection dose at 24 HPI. In contrast, *L. lactis* proliferated within the host for at least the first 24 h after infection where it reached an average load of between 100,000 and 1,000,000 CFU per fly. In the absence of host defense against the bacteria, this suggests that between 6 and 9 rounds of bacterial replication took place during this 24‐h period in flies infected with *L. lactis*. We also found striking variance in individual *L. lactis* loads, ranging from 0 to over one million bacteria per fly after 24 h. Although the cause of the variance in our population is unknown, Lazzaro et al. (2006) found that sequence polymorphisms and a deletion in *SR‐CII* intron 2 were associated with increased resistance to infection with *L. lactis*. It would be interesting to investigate whether we find a similar haplotype structure in *SR‐CII*.

Both bacteria species produced infections that were able to persist for at least 1 week, at which point the *L. lactis* load was reduced compared to 24 HPI but still around 5‐fold higher than the original inoculation level. Therefore, the immune system seems unable to clear either bacterial infection. Similar persistent infections have been found in the mealworm beetle, *Tenebrio molitor*, infected with the gram‐positive bacterium *Staphylococcus aureus* (Haine et al. 2008) and in *D. melanogaster* infected with the yeast *Candida glabrata* (Quintin et al. 2013).

The measured bacteria loads and the infections' persistence could result from a combination of host and parasite traits. For example, *E. coli* may have had lower loads because it failed to proliferate inside the host and/or may have been more efficiently kept in check by the host immune system. *E. coli* and *L. lactis* are targeted by different immune signaling pathways. The Imd pathway is activated by the presence of Gram‐negative bacteria, such as *E. coli*. Antimicrobial peptides (AMPs) elicited by this pathway show an acute expression profile, with peak expression occurring approximately 6 HPI (Lemaitre et al. 1997; Lemaitre and Hoffmann 2007; Broderick et al. 2009). In contrast, genes regulated by the Toll pathway tend to be activated by Gram‐positive bacteria, like *L. lactis*, and show peak expression 24 HPI (Lemaitre et al. 1997; Lemaitre and Hoffmann 2007; Broderick et al. 2009). There is evidence that these antimicrobial defenses can even act in the host insect hemolymph for weeks after infection, which is thought mainly to control persistent bacterial infections (Haine et al. 2008). Differences in the time post‐infection at which these signaling pathways are activated, and variability in longevity of the expression of different immune genes could contribute toward the disparity in bacteria load.

Yeast reduction had no effect on bacteria load in contrast to fecundity measures. Evidence for the effects of dietary protein on resistance and resource allocation in *D. melanogaster* is mixed (e.g., McKean and Nunney 2005; McKean et al. 2008), and the outcome seems to be dependent on the dietary component that is manipulated or on the bacteria species used for infections (McKean and Nunney 2005; McKean et al. 2008; Howick and Lazzaro 2014; Unckless et al. 2015). We may have found variation in resistance according to diet had the difference in yeast availability between our two treatments been greater. For example, McKean and Nunney (2005) found reduced *E. coli* load when females had *ad libitum* yeast conditions compared to standard food prior to infection. The individuals on SY did not have access to supplemental yeast in this study, so both of our dietary treatments (RY and SY) could be considered limited in comparison with McKean and Nunney's (2005) study. Nonetheless, under the conditions tested, resistance to both bacteria is unaffected by a 75% difference in dietary yeast concentration, in stark contrast to fecundity differences on the two diets.

### Tolerance varies according to infecting bacteria species

Dietary restriction uncovered variation in tolerance in a bacteria‐ and time‐dependent manner. Unexpectedly, individuals infected with *E. coli* on the RY medium appeared to be more tolerant to an infection than flies reared on SY medium 24 HPI, but individuals reared on RY medium became less tolerant to an infection 48 h later, suggesting that disease tolerance is a plastic rather than a static process that is influenced by the host environment. We note again that this effect was marginally significant. Mice infected with *Listeria monocytogenes*, a virulent bacterial pathogen, exhibited a similarly dynamic response, with tolerance being expressed differently as an infection progressed (Lough et al. 2015). Disease tolerance tended to improve in mice that survived the initial acute infection phase, which was characterized by pathogen proliferation, and increased resistance in survivors (Lough et al. 2015). Our study examines tolerance at two timepoints across a range of pathogen densities, where tolerance does indeed change at each timepoint in question. Bacterial infection in flies has likewise been characterized according to acute versus chronic infection stages (e.g., Howick and Lazzaro 2014). In this case, however, variation in tolerance (measured as mean fitness plotted against mean bacteria load, i.e., point tolerance) among different genotypes infected with *P. rettgeri* was most pronounced during the acute infection stage (i.e., the first 48 h post‐infection) rather than later in the infection (Howick and Lazzaro 2014). In consideration of these results, we suggest that host expression of tolerance seems to depend not only on host environment and time after infection but also on the particular pathogen. In flies*,* it seems that *E. coli* has the potential to affect tolerance despite the fact that we used an infection dose that is considerably lower than what has been shown to cause mortality in another fly genotype (1.85 × 10^5^ cells·mL^−1^; Ramsden et al. 2008). It might be worth considering that immune responses themselves can be costly because of self‐damage or autoreactivity (Sadd and Siva‐Jothy 2006; Haine et al. 2008; Stjernman et al. 2008; Bashir‐Tanoli and Tinsley 2014). If such factors increase as bacteria load increases, and if they have a negative impact on fecundity, it might help to explain the appearance of more negative tolerance slopes. As resistance was unaffected by dietary treatment in our model, while tolerance was, this may be evidence that the mechanisms underlying resistance and the mechanisms underlying tolerance are separate, at least in this scenario. Similarly, by manipulating glucose levels, Howick and Lazzaro (2014) uncovered significant genotype by diet effects on tolerance but not resistance to *P. rettgeri* during the first 48 h of an infection.

Despite high overall pathogen burdens, we did not detect differences in tolerance in flies infected with *L. lactis*. Although there was no effect of an infection at 24 and 72 HPI, it could be that host‐derived danger signals or damage signals caused by the bacteria's proliferation and subsequent persistence are not elicited until later in the infection and that pathogenic effects on fecundity, and tolerance, take more time to emerge (Lazzaro and Rolff 2011; Moreno‐García et al. 2014; Lough et al. 2015). Indeed, *L. lactis‐*infected flies showed reduced survival at 168 HPI, suggesting that the bacteria or the host immune system damages the host later in the infection. Damage sensing and danger signaling could play a central role in the mechanisms governing host immune strategies (Lazzaro and Rolff 2011; Moreno‐García et al. 2014).

## Conclusions

In contrast to previous studies in animals that focus on genotypic variation in resistance and tolerance (Råberg et al. 2007; Lefèvre et al. 2011; Vale and Little 2012; Howick and Lazzaro 2014; Jackson et al. 2014), we tested the effects of extrinsic factors on a single population (but see Sternberg et al. 2013; Hayward et al. 2014). We show that in the acute infection phase, *D. melanogaster* are able to maintain fitness despite high *L. lactis* loads and that there was no effect on tolerance despite dietary restriction. *E. coli* loads were orders of magnitude lower than *L. lactis*. In contrast to *L. lactis*‐infected flies, dietary restriction reduced viability in *E. coli*‐infected individuals and also affected tolerance. This indicates that diet can affect tolerance in a bacterium‐ and time‐dependent manner and that extrinsic factors, independent of genotype, can influence tolerance. To the best of our knowledge, this is the first study using *D. melanogaster* as a model to examine tolerance, where individual‐level rather than population means have been used to plot reaction norms. It might be interesting in future to apply tolerance estimates to flies at the individual level. The idea of individual‐level tolerance is relatively recent (Hayward et al. 2014; Lough et al. 2015) but studies on individual variation and intragenotype variability are essential to our understanding of host immune traits and may help to uncover the mechanistic basis underlying plasticity in host life history responses.

## Conflict of Interest

None declared.

## Supporting information


**Figure S1.** Fecundity 24 h post‐infection for females homogenized 24 h post‐infection only.

**Figure S2.** Average viability for files from each infection treatment and raised on RY (reduced yeast) and SY (standard yeast) diets.

**Figure S3.** Female survival for the second experiment over 168 h post infection.

**Table S1.** Parameter estimates corresponding to Models 1a, b and 2, Table 1.
**Table S2.** Parameter estimates corresponding to Models 3a, b, Table 2.
**Table S3.** Parameter estimates corresponding to Models 4a, b, Table 3.
**Table S4.** Parameter estimates corresponding to Models 5 and 6a, b, Table 4.
**Table S5.** The effect of infection status and diet on fecundity (*N* = 240) in flies homogenized at 24 HPI.

**Appendix S1.** Mating methods and results.
