# Supplementary figures and images for: The effect of diet and time after bacterial infection on fecundity, resistance, and tolerance in Drosophila melanogaster
Source: Ecol Evol. 2016 May 25;6(13):4229–42. doi: 10.1002/ece3.2185 (PMC4884575; doi:10.1002/ece3.2185)

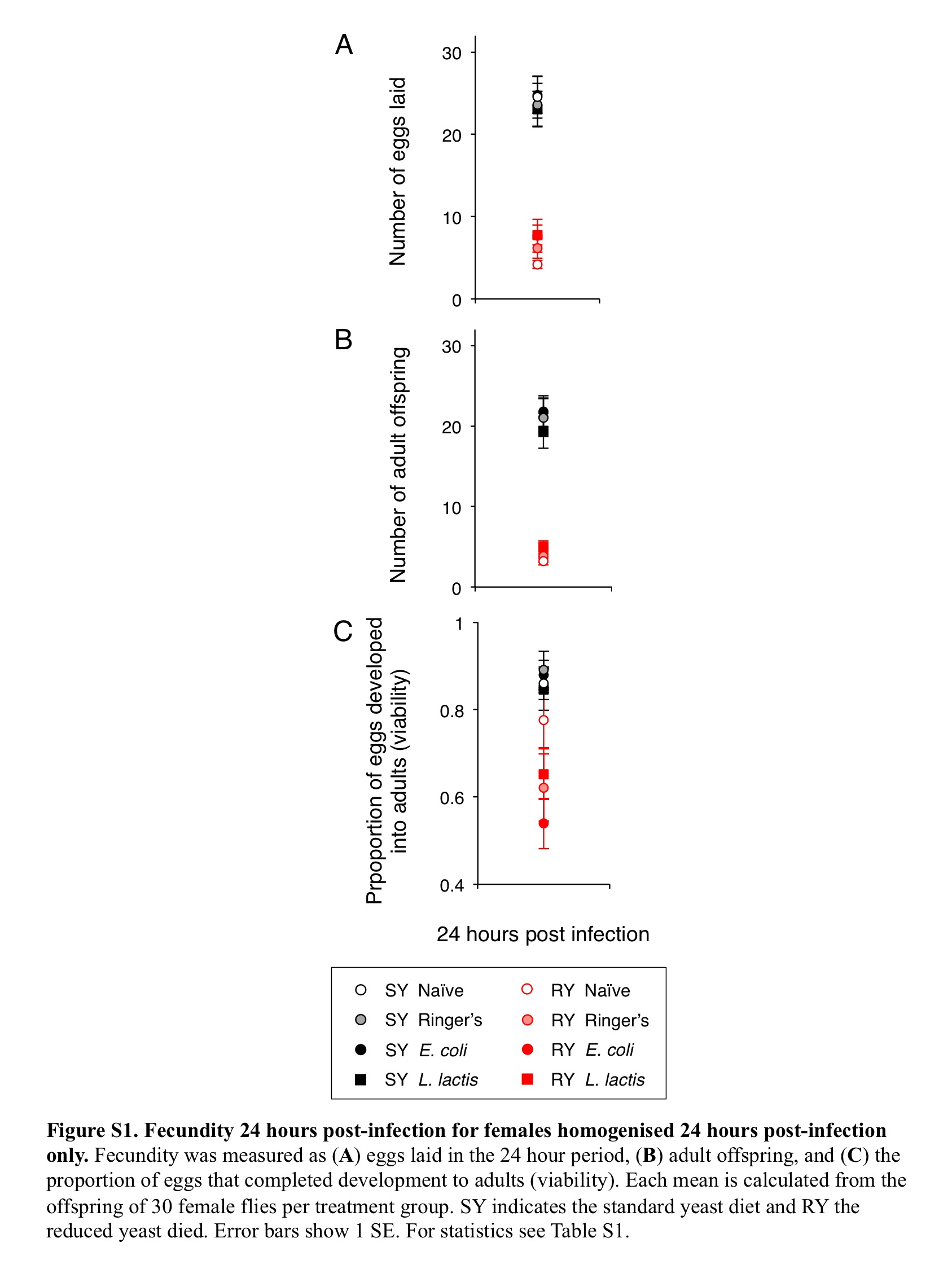

Supplement: Supplementary file 1 — Figure S1. Fecundity 24 h post‐infection for females homogenized 24 h post‐infection only. [file ECE3-6-4229-s001.tiff]

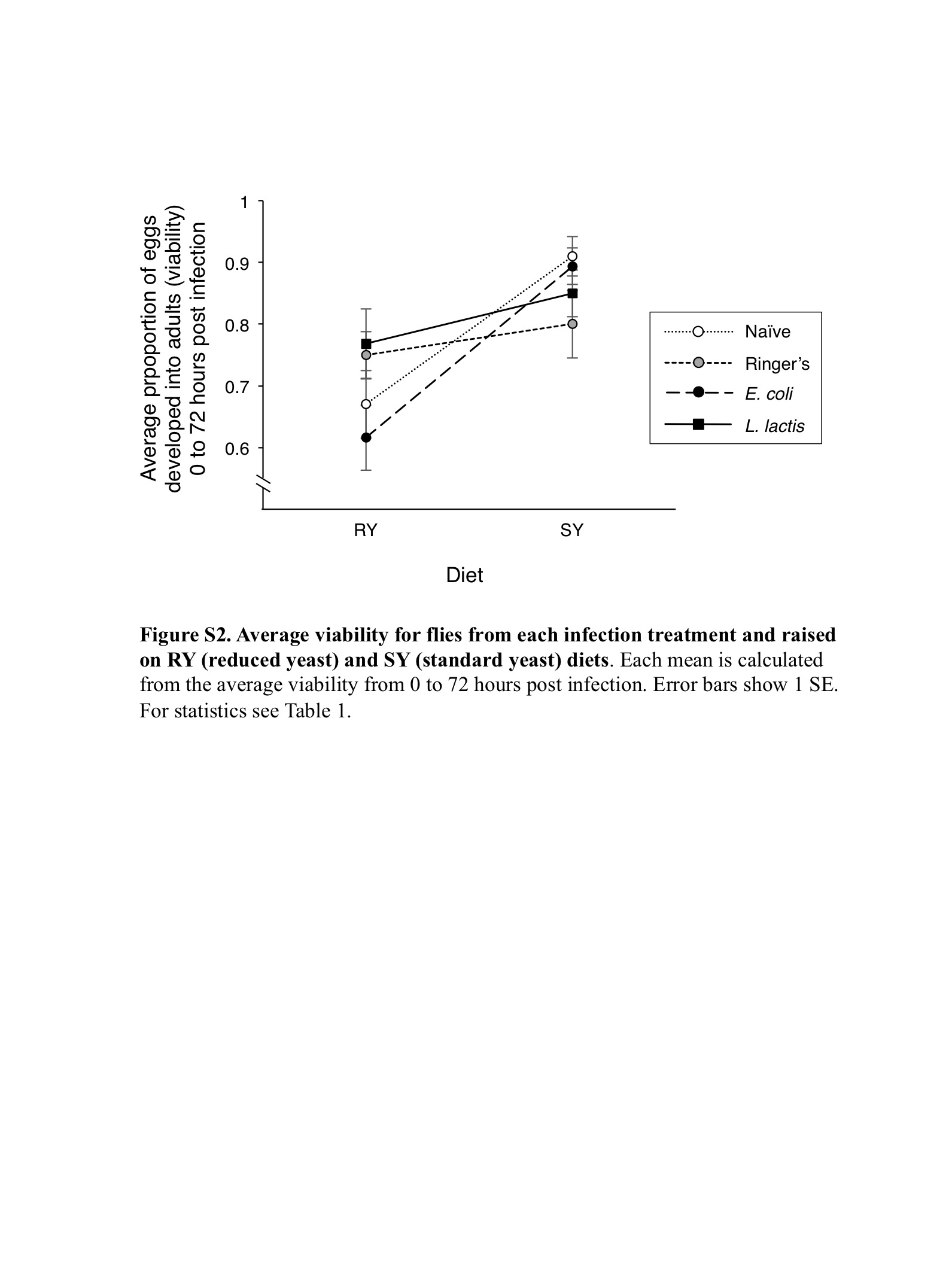

Supplement: Supplementary file 2 — Figure S2. Average viability for files from each infection treatment and raised on RY (reduced yeast) and SY (standard yeast) diets. [file ECE3-6-4229-s002.tiff]

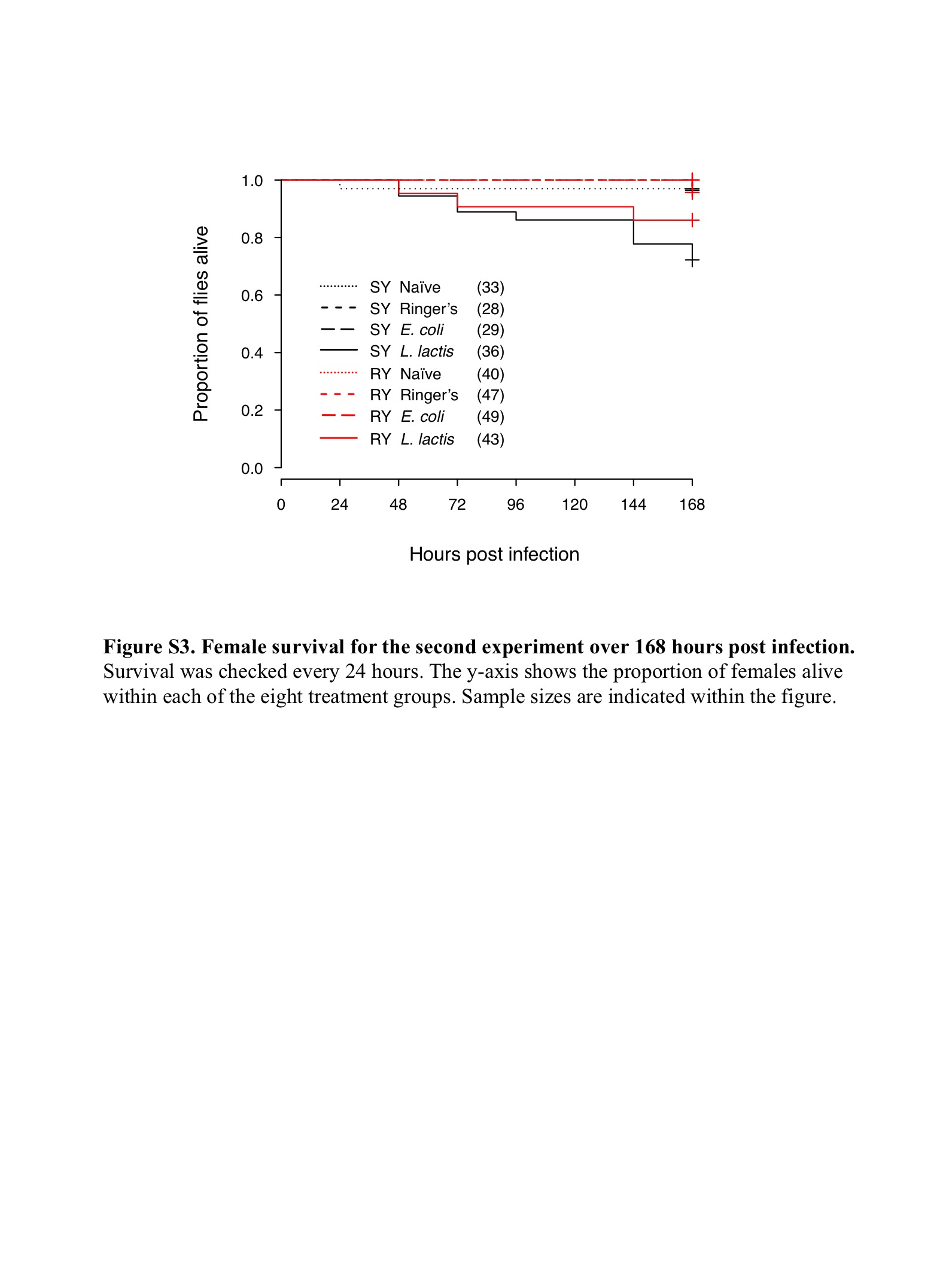

Supplement: Supplementary file 3 — Figure S3. Female survival for the second experiment over 168 h post infection. [file ECE3-6-4229-s003.tiff]
