# Supplementary material for: The effect of diet and time after bacterial infection on fecundity, resistance, and tolerance in Drosophila melanogaster
Source: Ecol Evol. 2016 May 25;6(13):4229–42. doi: 10.1002/ece3.2185 (PMC4884575; doi:10.1002/ece3.2185)
Supplement: Supplementary file 4 — Table S1. Parameter estimates corresponding to Models 1a, b and 2, Table 1. Table S2. Parameter estimates corresponding to Models 3a, b, Table 2. Table S3. Parameter estimates corresponding to Models 4a, b, Table 3. Table S4. Parameter estimates corresponding to Models 5 and 6a, b, Table 4. Table S5. The effect of infection status and diet on fecundity (N = 240) in flies homogenized at 24 HPI. [file ECE3-6-4229-s004.docx]

**Table S1. Parameter estimates corresponding to Models 1a, b and 2, Table 1.**

|  | Model 1a: Eggs | | Model 1b: Offspring | | Model 2: Viability | |
| --- | --- | --- | --- | --- | --- | --- |
|  | *Estimate* | *Std. Error* | *Estimate* | *Std. Error* | *Estimate* | *Std. Error* |
| **Intercept** | 3.169 | 0.115 | 2.905 | 0.136 | 1.745 | 0.287 |
| **Infection** |  |  |  |  |  |  |
| Naïve | -0.112 | 0.142 | -0.007 | 0.168 | 0.988 | 0.391 |
| *E. coli* | -0.051 | 0.142 | -0.031 | 0.168 | 0.949 | 0.396 |
| *L. lactis* | -0.176 | 0.143 | -0.123 | 0.168 | 0.302 | 0.390 |
| **Diet** |  |  |  |  |  |  |
| RY | -1.691 | 0.104 | -1.922 | 0.123 | -0.492 | 0.434 |
| **Time** |  |  |  |  |  |  |
| 48 | -0.263 | 0.111 | -0.248 | 0.091 | 0.009 | 0.144 |
| 72 | -0.806 | 0.116 | -0.835 | 0.099 | -0.232 | 0.152 |
| **Infection x Diet** |  |  |  |  |  |  |
| Naïve x RY | - | - | - | - | -1.428 | 0.584 |
| *E. coli* x RY | - | - | - | - | -1.578 | 0.583 |
| *L. lactis* x RY | - | - | - | - | -0.14 | 0.590 |
| **Infection x HPI** |  |  |  |  |  |  |
| Naïve x 48 | 0.283 | 0.144 | 0.216 | 0.119 | - | - |
| *E. coli* x 48 | -0.039 | 0.145 | -0.064 | 0.122 | - | - |
| *L. lactis* x 48 | -0.007 | 0.146 | 0.026 | 0.122 | - | - |
| Naïve x 72 | 0.275 | 0.153 | 0.267 | 0.130 | - | - |
| *E. coli* x 72 | 0.222 | 0.152 | 0.252 | 0.129 | - | - |
| *L. lactis* x 72 | 0.000 | 0.156 | 0.063 | 0.134 | - | - |
| **Diet x HPI** |  |  |  |  |  |  |
| RY x 48 | 0.564 | 0.106 | 0.652 | 0.093 | 0.528 | 0.240 |
| RY x 72 | 0.373 | 0.113 | 0.532 | 0.103 | 0.816 | 0.268 |

**Table S2. Parameter estimates corresponding to Models 3a, b, Table 2.**

|  | Model 3a: *E. coli* | | Model 3b: *L. lactis* | |
| --- | --- | --- | --- | --- |
|  | *Estimate* | *Std. Error* | *Estimate* | *Std. Error* |
| **Intercept** | 6.709 | 0.253 | 8.808 | 0.419 |
| **Diet** |  |  |  |  |
| RY | -0.105 | 0.239 | 0.205 | 0.701 |
| **HPI** |  |  |  |  |
| 72 | -0.778 | 0.220 | 1.494 | 0.409 |
| **Diet x HPI** |  |  |  |  |
| RY x 72 | 0.221 | 0.354 | -0.524 | 0.872 |

**Table S3. Parameter estimates corresponding to Models 4a, b, Table 3.**

|  | Model 4a: *E. coli* | | Model 4b: *L. lactis* | |
| --- | --- | --- | --- | --- |
|  | *Estimate* | *Std. Error* | *Estimate* | *Std. Error* |
| **Intercept** | 119.891 | 87.684 | 0.9366 | 35.407 |
| **CFU** | -17.342 | 12.915 | -1.459 | 3.858 |
| **Diet** |  |  |  |  |
| RY | -255.348 | 119.524 | 44.498 | 20.434 |
| **HPI** |  |  |  |  |
| 72 | 24.317 | 107.312 | -20.445 | 57.989 |
| **Diet x HPI** |  |  |  |  |
| RY x 72 | 347.891 | 167.702 | -87.089 | 27.913 |
| **CFU x Diet** |  |  |  |  |
| CFU x RY | 39.475 | 17.761 | - | - |
| **CFU x HPI** |  |  |  |  |
| CFU x 72 | -2.083 | 16.661 | 4.426 | 5.708 |
| **CFU x Diet x HPI** |  |  |  |  |
| CFU x 72 x RY | -59.033 | 26.241 | - | - |

**Table S4. Parameter estimates corresponding to Models 5 and 6a, b, Table 4.**

|  | Model 5: Protein | | Model 6a: *E. coli* | | Model 6b: *L. lactis* | |
| --- | --- | --- | --- | --- | --- | --- |
|  | *Estimate* | *Std. Error* | *Estimate* | *Std. Error* | *Estimate* | *Std. Error* |
| **Intercept** | 11.774 | 0.666 | 3.846 | 0.209 | 8.505 | 0.201 |
| **Diet** |  |  |  |  |  |  |
| RY | -0.848 | 0.372 | 0.429 | 0.385 | -0.207 | 0.66 |
| **Infection** |  |  |  |  |  |  |
| Naïve | -0.476 | 0.524 | - | - | - | - |
| *E. coli* | -0.696 | 0.515 | - | - | - | - |
| *L. lactis* | 0.223 | 0.522 | - | - | - | - |

|  | Eggs | | Offspring | | Egg viability | |
| --- | --- | --- | --- | --- | --- | --- |
| *Tested effect* | *χ^2^* | *P* | *χ^2^* | *P* | *χ^2^* | *P* |
| Infection status | 4.1 | 0.25 | 2.12 | 0.55 | 0.69 | 0.8754 |
| Diet | 166.86 | **< 0.0001** | 205.86 | **< 0.0001** | 21.49 | **< 0.0001** |

**Table S5. The effect of infection status and diet on fecundity (N=240) in flies homogenized at 24 HPI.** We started by testing interations between infection status and diet, and model reduction was performed as described in the materials and methods. We used the following final model for each variable tested: Eggs/ Offspring/ Egg viability ~ Infection status + Diet + Replicate/Block/FlyID_random_.
