# Supplementary material for: The effect of diet and time after bacterial infection on fecundity, resistance, and tolerance in Drosophila melanogaster
Source: Ecol Evol. 2016 May 25;6(13):4229–42. doi: 10.1002/ece3.2185 (PMC4884575; doi:10.1002/ece3.2185)
Supplement: Supplementary file 5 — Appendix S1. Mating methods and results. [file ECE3-6-4229-s005.docx]

Appendix S1. Mating methods and results.

*Experiment 1: Effect of diet on mating duration and mating latency, effect of mating duration on adult offspring number or egg number*

We began by examining the effects of diet on mating duration (MD) and mating latency (ML), as well as the effect of mating duration on total egg or offspring numbers over the three-day experimental period, using generalized linear mixed models in the lme4 (Supplementary Model 1) and MASS (Model 2) packages with Poisson error structures. FlyID nested within replicate were included as random effects.

Supplementary Model 1a, b: MD or ML ~ Diet + Replicate/FlyID_random_

Supplementary Model 2a, b*:* Total Eggs or Total Offspring ~ MD + Replicate/FlyID_random_

*Results: Longer mating duration resulted in higher fecundity*

We found no effect of diet on mating latency (z = -1.304, p = 0.192) or copulation duration (z = 1.07, p = 0.284). Mating latency (± 1 standard error) was 35.98 ± 2.26 minutes for individuals on the reduced yeast diet (RY) and 31.15 ± 1.85 minutes for individuals on standard yeast diet (SY). The mean copulation duration for individuals on RY was 19.16 ± 0.47 minutes and 19.81 ± 0.48 minutes for flies on SY. However, copulation duration positively affected total egg number (t = 2.15, p = 0.032) and total offspring number (t = 2.24, p = 0.026) in this wild type fly line.

Our finding that dietary protein manipulation did not affect copulation duration and mating latency is similar to what has been found in the *D. melanogaster* Dahomey wild type when male dietary protein was manipulated (Fricke, Bretman & Chapman, 2008). The average mating duration of our wild type fly population is similar to what has been found in the Dahomey wild type, but the mating latency was around 50 % longer in our wild type fly line (Bretman, Westmancoat & Chapman, 2013). Interestingly, we found that a longer copulation duration correlated with increased eggs and adult offspring, whether this is a result of e.g., more sperm or accessory gland products being transferred remains to be tested.

**References**

Bretman, A., Westmancoat, J.D. & Chapman, T. (2013) Male control of mating duration following exposure to rivals in fruitflies. *Journal of Insect Physiology,* 59, 824-827.

Fricke, C., Bretman, A. & Chapman, T. (2008) Adult male nutrition and reproductive success in *Drosophila melanogaster. Evolution,* 62, 3170-3177.
